# Regulation of stomatal development by receptor-like kinases and receptor-like proteins in *Arabidopsis* and grasses

**DOI:** 10.3389/fpls.2026.1770946

**Published:** 2026-02-26

**Authors:** Wenqi Zhou, Wenjin Wang, Mingfeng Zhao, Yongsheng Li, Haijun He, Yuqian Zhou

**Affiliations:** 1 Maize Research Center of Gansu Province, Crop Research Institute, Gansu Academy of Agricultural Sciences, Lanzhou, China; 2 Key Laboratory of Cell Activities and Stress Adaptations, Ministry of Education, Lanzhou University, Lanzhou, China; 3 Key Laboratory of Gene Editing for Breeding, School of Life Sciences, Lanzhou, China

**Keywords:** *Arabidopsis*, LRR-RLKs, LRR-RLPs, signal cascade, stomatal development, subsidiary cell

## Abstract

Stomata are core channels for plant gas exchange and water transpiration, and precise regulation of their development directly impacts photosynthetic efficiency, water use, and stress resistance. Plant receptor kinases, particularly leucine-rich repeat receptor-like kinases (LRR-RLKs), function as key signal sensors: they perceive endogenous and exogenous signals, trigger downstream cascades, and finely regulate stomatal initiation, differentiation, and patterning. Deciphering these mechanisms is therefore critical for improving crop stomatal traits, stress tolerance, and yield. Among receptors regulating stomatal development, LRR-RLKs and leucine-rich repeat receptor-like proteins (LRR-RLPs) are the best studied. Too Many Mouths (TMM), the first identified stomatal receptor (LRR-RLP), forms a multiprotein complex with ERECTA family (ERf) and SERK family LRR-RLKs. This complex recognizes Epidermal Patterning Factors (EPFs)/EPF-like factors (EPFLs) peptides., activates the YODA (YDA)-MAPK cascade, and inhibits the key stomatal lineage transcription factor SPEECHLESS (SPCH), thereby precisely regulating stomatal patterning and differentiation in *Arabidopsis*. Beyond this core complex, other LRR-RLKs (e.g., HSL1, CLV1, MUS) also regulate *Arabidopsis* stomatal development or morphogenesis. HSL1 recruits SERK co-receptors and perceives CLE9/10 ligands, but the ligands for CLV1 and MUS in stomatal development remain unknown. Notably, maize PAN1 and PAN2 are essential for asymmetric division of subsidiary mother cells (SMCs) and subsidiary cell (SC) formation, with their cognate ligands also uncharacterized. This review summarizes key advances in stomatal receptor (especially LRR-RLK) mediated stomatal development in *Arabidopsis* and grasses and highlights core issues such as ligand recognition.

## 1 Introduction: an overview of stomatal development and its genetic control

Stomata represent one of the earliest specialized structures to evolve in plants following their transition from aquatic to terrestrial habitats. Widely distributed across the epidermis of aerial plant tissues and organs, stomata are pivotal for plants to adapt to the fluctuating natural environment (Nadeau and Sack, 2002a). The stomata of most plant species consist of a pair of kidney-shaped guard cells (GCs), whereas those of grasses are composed of a pair of dumbbell-shaped GCs flanked by two triangular or dome-shaped subsidiary cells (SCs). By opening and closing dynamically, stomata regulate the uptake of carbon dioxide as well as the release of water vapor and oxygen, thereby serving as a critical gateway for gas exchange between plants and the external environment and governing plant photosynthesis and transpiration (Bergmann and Sack, 2007). Stomata also function as major entry points for pathogenic microorganisms into plant tissues. Upon detecting pathogenic bacteria, stomata rapidly close to restrict pathogen invasion, thus playing a key role in plant innate immunity (Han et al., 2021; Hou et al., 2024).

EPF/EPFL family peptides (EPF1/2, EPFL4/5/6, EPFL9) and CLE9/10 serve as extracellular signals, which are perceived by plasma membrane receptor complexes-primarily the TMM-ERf (ER/ERL1/ERL2)-SERK and HSL1-SERK complexes-with additional signaling input from the CLV1-MAZ and MUS pathways. Activated receptors trigger a phosphorylation cascade (YODA → MKK4/5/7/9 → MPK3/6), which transduces signals into the nucleus where MPK3/6 phosphorylates key transcription factors. This constitutes the central signaling network of stomatal development, which integrates various plant hormone signals and environmental stimulus. The phytohormone signals and environmental cues integrated by the YDA-MAPK cascade influence each step of stomatal development. The SPCH-SCRM complex drives the differentiation of protodermal cells (purple) into meristemoid mother cells (MMCs, pale yellow) and orchestrates MMC asymmetric division to form meristemoids (Ms, yellow). MUTE-SCRM mediates the transition of Ms into guard mother cells (GMCs, orange), while FAMA-SCRM directs GMC symmetric division to generate mature guard cells (GCs, green). FLP also modulates the Ms-to-GMC and GMC-to-GC differentiation processes. The section below the dashed line depicts the stomatal developmental process and the spatiotemporal expression patterns of RLPs and RLKs (colors correspond to those of plasma membrane receptors). This temporal regulation drives the asymmetric division of purple MMCs, subsequent symmetric division of orange GMCs, and the ultimate formation of green mature GCs. Pathways marked with “?” (e.g., the downstream signaling of CLV1-MAZ and MUS) represent under-characterized molecular mechanisms that remain active areas of research. Yellow arrows indicate feedback regulatory loops.

**Figure 1:**
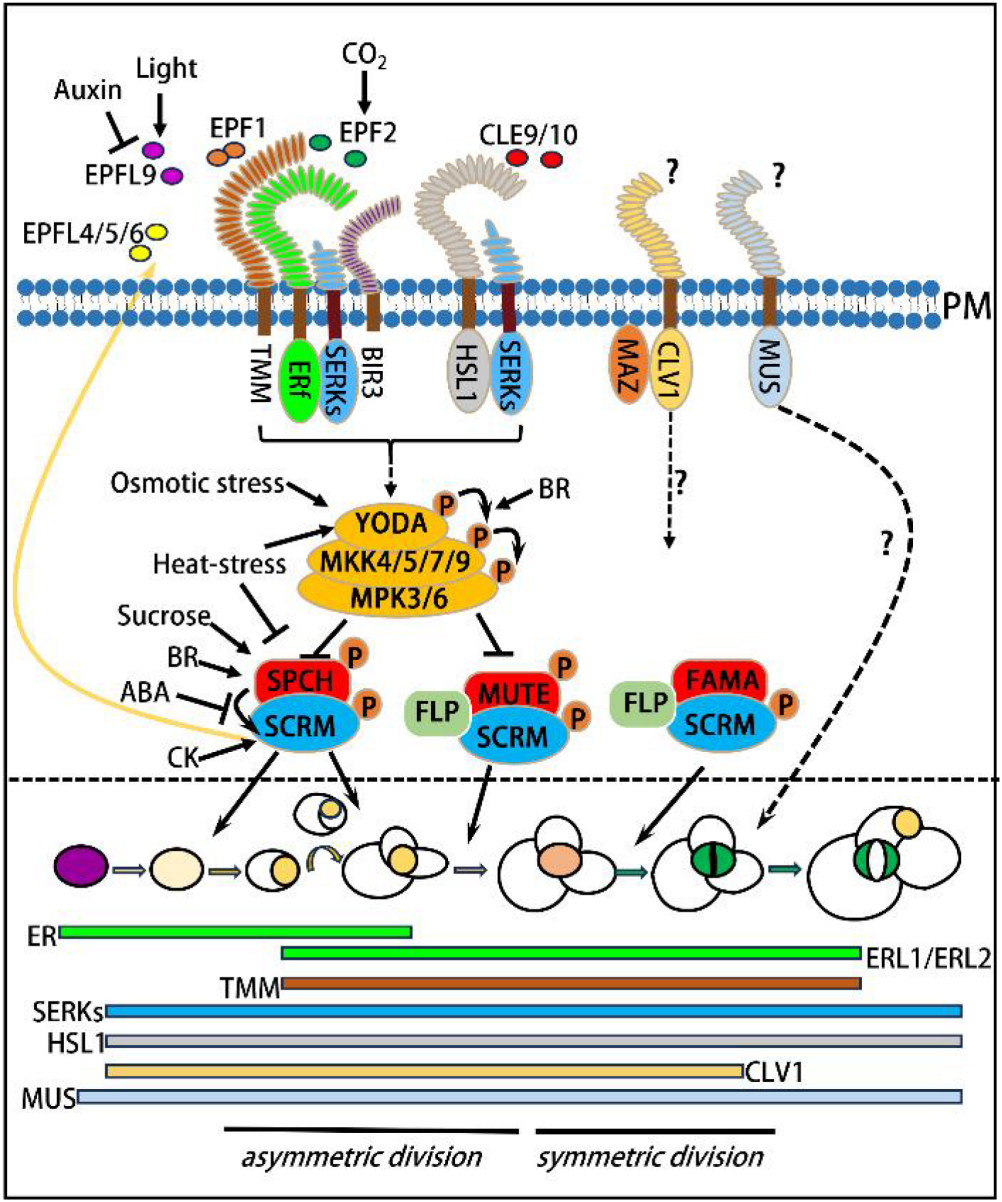
Schematic model of the peptide-receptor signaling network regulating stomatal development in *Arabidopsis*.

**Figure 2:**
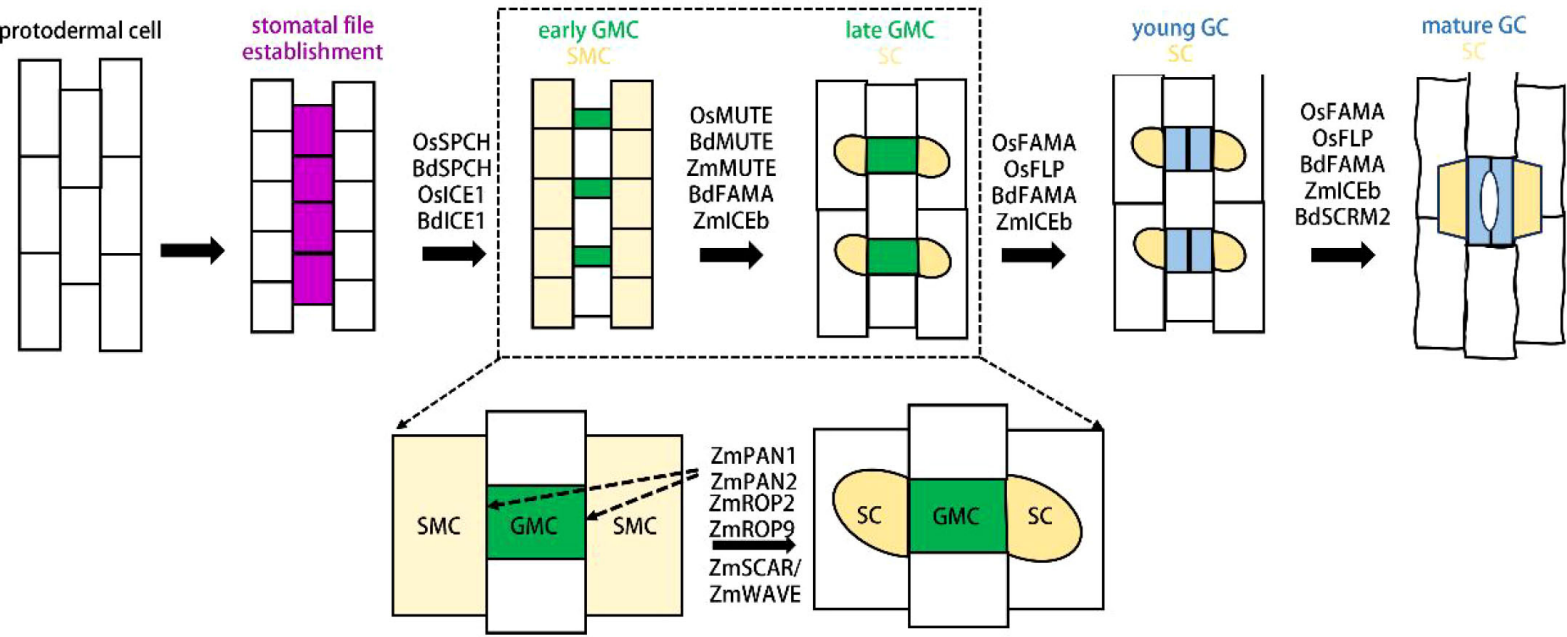
The molecular of stomatal development in grasses.

Stomatal development in *Arabidopsis* begins with some protodermal cells acquiring the fate of meristemoid mother cells (MMCs), which subsequently undergo asymmetric division to produce a small meristemoid (M) and a large stomatal lineage ground cell (SLGC). The M possesses stem cell activity and can undergo 0 to 3 rounds of asymmetric division, generating new M cells and additional SLGCs; eventually, the M cell differentiates into a guard mother cell (GMC). The GMC undergoes one round of symmetric division to form a pair of guard cells (GCs), which further undergo morphogenesis to form a mature stomatal complex consisting of a pair of kidney-shaped GCs. Regarding SLGCs, they have two possible developmental fates: first, directly differentiating into pavement cells; second, undergoing asymmetric division at positions far from stomata or stomatal precursor cells to produce satellite Ms, which eventually form stomata. This process follows the “one-cell spacing rule” (**Figure 1**). In gramineous plants (taking *Oryza sativa* as an example), stomatal development initiates at the base of the leaf: specific rows of protodermal cells on both sides of the leaf veins first acquire the fate of stomatal lineage cells and undergo one asymmetric division toward the leaf tip to produce a GMC. After maturation, the GMC induces adjacent subsidiary mother cells (SMCs) to undergo one asymmetric division to form subsidiary cells (SCs). Subsequently, the GMC undergoes one round of symmetric division to generate a pair of GCs, which further undergo morphogenesis to form a mature stomatal complex composed of a pair of dumbbell-shaped GCs and a pair of triangular SCs located on either side of the GCs. (**Figure 2**) (Nadeau and Sack, 2002b; Bergmann and Sack, 2007; Lau and Bergmann, 2012; Pillitteri and Torii, 2012; Pillitteri and Dong, 2013; Kim and Torii, 2024; Chen, 2024; Zhou et al., 2024; Berg and Raissig, 2025).

The protodermal cell rows on both sides of leaf veins acquire stomatal lineage fate (purple). Cells within these rows undergo asymmetric division to generate early guard mother cells (GMCs, green); upon maturation, GMCs induce the subsidiary mother cells (SMCs, pale yellow) on both sides to undergo asymmetric division, producing subsidiary cells (SCs, yellow). Subsequently, GMCs undergo symmetric division to form a pair of guard cells (GCs, blue). The lower panel shows that receptor-like kinases ZmPAN1/2 interact with ZmROP2/9 and the SCAR/WAVE complex to regulate the asymmetric division of SMCs in maize. Dashed arrows indicate the localization of ZmPAN1/2 at the contact interface between GMCs and SMCs.

Notably, the formation of stomata in *Arabidopsis* is mainly regulated by three sequentially and specifically expressed basic helix-loop-helix (bHLH)Ia family transcription factors, namely SPCH, MUTE, and FAMA (Ohashi-Ito and Bergmann, 2006; MacAlister et al., 2007; Pillitteri et al., 2007, Pillitteri et al., 2008). These factors form complexes with bHLH IIIb family transcription factors SCRMs (including ICE1/SCRM1 and SCRM2) to exert their regulatory functions (Kanaoka et al., 2008). Specifically, the SPCH-SCRM complex promotes the differentiation of protodermal cells into MMCs and regulates the asymmetric division of MMCs to generate Ms; the MUTE-SCRM complex mediates the transformation of Ms into GMCs; and the FAMA-SCRM complex controls the symmetric division of GMCs to produce a pair of GCs. In addition, the MYB transcription factor FLP is also involved in regulating the differentiation of Ms into GMCs and GMCs into GCs (**Figure 1**) (Lai et al., 2005; Lee et al., 2014; Li et al., 2023, Li et al., 2025). Similarly, stomatal differentiation in gramineous plants is primarily controlled by homologous genes of these transcription factors. The OsSPCH-OsICE1 and BdSPCH-BdICE1 complexes regulate the initiation of stomatal development, and loss-of-function mutations in these genes result in a complete absence of stomata (Liu et al., 2009; Raissig et al., 2016; Wu et al., 2019). OsMUTE, BdMUTE, and ZmMUTE promote GMC differentiation and can move from GMCs to adjacent SMCs to induce their asymmetric division for SC formation (Raissig et al., 2017; Wang et al., 2019; Wu et al., 2019). OsFAMA, BdFAMA, and BdSCRM2 regulate GC differentiation following GMC symmetric division; loss-of-function mutations in these genes lead to four-celled complexes arrested post-GMC symmetric division (Raissig et al., 2016; Wu et al., 2019). Additionally, BdFAMA exhibits functional redundancy with BdMUTE in promoting GMC differentiation (McKown et al., 2023). Furthermore, OsFLP also regulates the differentiation of GMCs into GCs; its loss-of-function leads to partial GMC arrest post-symmetric division and abnormal division directions in some GMCs. ZmICEb mainly modulates GMC differentiation, SC formation, and GC maturation (**Figure 2**) (Wu et al., 2019; Zhang et al., 2022; Chen et al., 2025; Zhou et al., 2024; Zhou et al., 2025).

Studies on *Arabidopsis* stomatal development have identified SPCH as the most upstream switch in the stomatal development pathway, capable of activating the expression of itself and SCRMs (MacAlister et al., 2007; Lau et al., 2014; Horst et al., 2015). Upon forming a complex, SPCH and SCRMs further enhance their own expression, establishing a positive feedback loop that promotes stomatal formation (Horst et al., 2015). Concurrently, the SPCH-SCRM complex activates specific stomatal ligands and receptors, which in turn trigger the downstream YODA-Mitogen-Activated Protein Kinase (MAPK) cascade. This cascade ultimately phosphorylates SPCH and SCRMs, leading to their degradation and the formation of a long-range negative feedback loop that inhibits stomatal formation (Horst et al., 2015; Ding et al., 2025). The interplay between these positive and negative feedback loops ultimately ensures the establishment of the “one non-stomatal cell spacing” pattern mentioned earlier. (**Figure 1**).

Plant hormones and environmental signals act on the epidermis to regulate stomatal development by modulating key components of the stomatal signaling pathway (Chen et al., 2020; Chen, 2023, Chen, 2024; Chua and Lau, 2024; Giannoutsou et al., 2025). Auxin levels decline dynamically in meristemoid cells, and this process is essential for GMC fate specification (Le et al., 2014). Auxin inhibits stomatal production by suppressing the expression of EPFL9/STOMAGEN, a positive regulatory ligand in stomatal development, and this inhibition is mediated by the auxin response factor 5 (ARF5)/MONOPTEROS (MP). Cytokinin (CK) induces SPCH expression, and experimental modulation of CK levels alters epidermal cell division patterns and stomatal production (Vatén et al., 2018). Abscisic acid (ABA) promotes the phosphorylation of SPCH in stomatal precursor cells, leading to its proteasomal degradation and the consequent suppression of stomatal formation (Yang et al., 2022). Brassinosteroid (BR) regulates stomatal development by inactivating BR INSENSITIVE2 (BIN2), its central signaling component. BIN2 phosphorylates SPCH to target it for degradation (Gudesblat et al., 2012) and also activates the YDA–MAPK cascade through the direct phosphorylation of YDA and MKK4 (Kim et al., 2012; Khan et al., 2013).

Light signals promote stomatal formation by inhibiting the RING E3 ubiquitin ligase CONSTITUTIVELY PHOTOMORPHOGENIC 1 (COP1), which represses stomatal development in darkness via the direct ubiquitination and degradation of SCRM (Kang et al., 2009; Lee et al., 2017). Additionally, red light induces the expression of B-GATA transcription factors, which directly bind to the SPCH promoter and activate its transcription (Klermund et al., 2016). Elevated CO_2_ levels suppress stomatal formation primarily by upregulating CO_2_ RESPONSIVE SECRETED PROTEASE (CRSP), which facilitates the maturation of Epidermal Patterning Factor 2 (EPF2) (Engineer et al., 2014). Under heat stress, the accumulation of PHYTOCHROME-INTERACTING FACTOR 4 (PIF4) leads to the transcriptional repression of SPCH; concurrently, heat stress enhances HSP90 activity, which potentiates the YDA–MAPK signaling cascade (Lau et al., 2018; Samakovli et al., 2020). Osmotic stress similarly reduces SPCH protein abundance by activating the YDA–MAPK cascade (Kumari et al., 2014). In contrast, sucrose promotes stomatal initiation by stabilizing SPCH through site-specific phosphorylation (Han et al., 2020).

## 2 TMM, the first identified stomatal receptor

**Figure 3:**
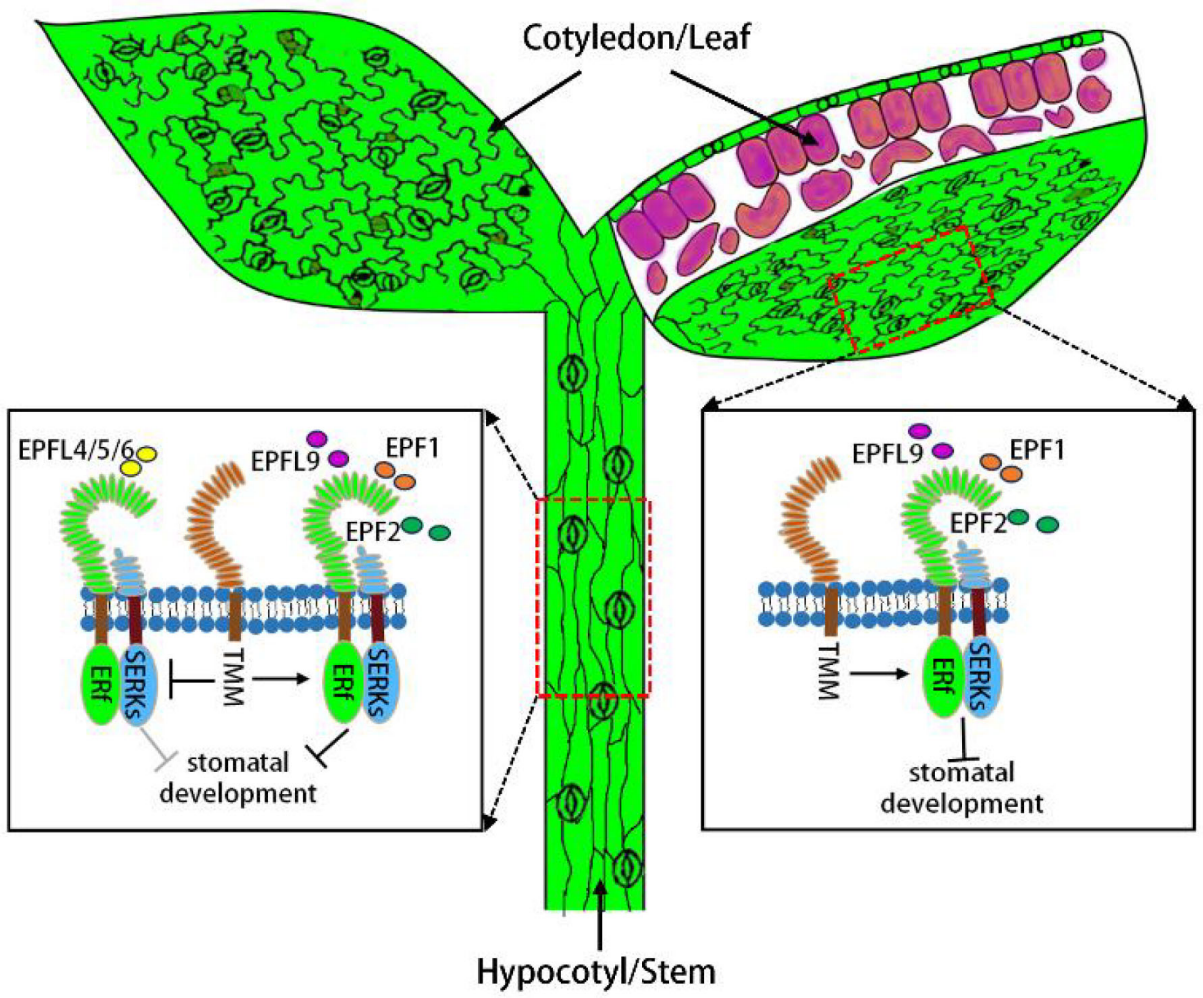
Genetic interactions of TMM with ERf and SERK receptors during stomatal development in cotyledon/leaf and hypocotyl/stem epidermis.

To identify the genes regulating stomatal development, Fred D. Sack’s research group was the first to screen EMS-mutagenized *Arabidopsis* mutants, yielding two epidermal stomatal clustering mutants designated too many mouths (*tmm*) and four lips (*flp*) (Yang and Sack, 1995). In the *tmm* mutant, neighboring cells of stomata or stomatal precursors undergo ectopic asymmetric division adjacent to stomata or their precursors, ultimately forming stomatal clusters (**Table 1**). This indicates that TMM modulates oriented asymmetric division to ensure the minimal one-celled spacing pattern between stomata (Geisler et al., 2000). Notably, in contrast to the leaf epidermis, the hypocotyl and stem epidermis of the *tmm* mutant fail to produce stomata (Geisler et al., 1998; Bhave et al., 2009), suggesting that TMM may exert distinctly different roles in regulating stomatal development in the epidermis of leaves versus hypocotyls/stems (Herrmann and Torii, 2021) (**Figure 3**). TMM encodes a LRR-RLP and is specifically expressed in proliferative protodermal cells and stomatal lineage cells (Nadeau and Sack, 2002a). The TMM protein contains 10 uninterrupted LRRs-domains that facilitate protein-protein or protein-ligand interactions-and a putative carboxyl-terminal (COOH-terminal) transmembrane domain. Since TMM lacks a cytoplasmic kinase domain, how does it transduce extracellular signals recognized by its LRR motifs across the membrane into the cell? It is hypothesized that TMM may function within a receptor complex containing LRR-RLKs (**Figure 1**) (Nadeau and Sack, 2002a; Lin et al., 2017).

**Table 1:** Overview of RLKs and RLPs involved in stomatal development.

Receptor class	Receptor types	Ligands	Interacting partners	Downstream signaling components	Cell-type specificity	Mutant phenotypes	Species
TMM	RLP		ERf,SERKs	MAPK cascade	M,GMC,immature GC	Stomatal clusters on leaves;no stomata on stems	*A. thaliana*
ERf	RLK	EPF1,EPF2,EPFL9, EPFL4/6	TMM,SERKs	MAPK cascade	M,GMC,immature GC	Stomatal clusters in ER, ERL1, ERL2 triple mutants	*A. thaliana*
SERKs	RLK	EPF1,EPF2,EPFL9	ERf,TMM, BIR3	MAPK cascade	Widely expressed in leaf epidermis	Stomatal clusters in SERK1,SERK2,BAK1,SERK4 quadruple mutants	*A. thaliana*
HSL1	RLK	CLE9/10	SERKs	MAPK cascade	Widely expressed in leaf epidermis	Both GCs and NGCs numbers were higher	*A. thaliana*
CLV1	RLK	Un known	MAZ	MAZ	Stomatal lineage non-differentiated cells	Stomatal clusters in true leaves	*A. thaliana*
MUS	RLK	Un known			cell plates in all epidermal divisions	Skewed stomatal pores; GCs with disrupted bilateral symmetry	*A. thaliana*
PAN1/2	RLK	Un known	ROP2/9, SCAR/WAVE		SMC membrane at SMC-GMC contact interface	Abnormal subsidiary cells	*Z.mays*

In the hypocotyl/stem epidermis, TMM promotes stomatal development by inhibiting ER-family genes’ negative effect (grey “T” shape). In the cotyledon/leaf epidermis, TMM induces ERf-SERK receptor complex formation to repress stomatal development. Upper left: leaf surface; Upper right: leaf cross section (purple: mesophyll cells); Lower: leaf hypocotyl.

## 3 ERf receptor-like kinases associate with TMM to regulate stomatal development

In *Arabidopsis*, LRR-RLK *ER* and its functional paralogs *ERL1* and *ERL2* act synergistically to regulate stomatal patterning and differentiation (Shpak et al., 2005; Lee et al., 2012; Lin et al., 2017). Both *ERL1* and *ERL2* were highly expressed in stomatal-lineage cells (**Table 1**) (Shpak et al., 2005). Single *er* mutants exhibit excessive division of stomatal-lineage cells and increased meristemoid formation. Introduction of an additional *erl2* mutation enhances the stomatal phenotype of *er*, whereas an additional *erl1* mutation promotes stomatal production (Shpak et al., 2005). However, loss-of-function mutations in all three genes (*er erl1 erl2*) result in the formation of large stomatal clusters. Genetic interactions between the ER family and TMM are dynamic (Shpak et al., 2005). In the stem epidermis, similar to *tmm*, *tmm er* and *tmm erl2* double mutants fail to produce stomata, indicating that *TMM* is epistatic to *ER* and *ERL2* (Shpak et al., 2005). By contrast, the *tmm erl1* double mutant partially restores stomatal production in the stem, suggesting partial epistasis of *TMM* to *ERL1*. Notably, the *tmm er erl1* triple mutant fully restores stomatal production in the stem epidermis, while the *tmm er erl1 erl2* quadruple mutant forms stomatal clusters identical to those observed in *er erl1 erl2* triple mutants. These findings indicate that *ER* family (*ERf*) genes are epistatic to *TMM*. Importantly, the epistasis of *TMM* to *ER* and *ERL2* is dependent on functional *ERL1*, implying that TMM may primarily suppress ERL1 activity to repress stomatal development (Shpak et al., 2005). In leaves, *tmm er* and *tmm er erl2* mutants produce stomatal lineage ground cells (SLGCs) but no mature stomata, suggesting that TMM is required for stomatal formation in the absence of ER. However, introduction of an additional *erl1* mutation into both *tmm er* and *tmm er erl2* backgrounds promotes stomatal differentiation, further supporting the notion that TMM inhibits ERL1 activity (Shpak et al., 2005). These complex interactions between *TMM* and *ER* family genes suggest that TMM and ERf receptors may act closely together, and ERf receptors are likely the candidate partners facilitating TMM-recognized signal transduction (Giannoutsou et al., 2025). Consistent with this hypothesis, previous studies have demonstrated that TMM forms constitutive complexes with ER or ERL1. Additionally, ERf receptors can form homodimers or heterodimers, whereas TMM does not associate with itself *in vivo* (**Figure 1**) (Lee et al., 2012; Lin et al., 2017; Herrmann and Torii, 2021).

## 4 SERK family receptor-like kinases form a multiprotein receptorsome with TMM and ERf receptors to regulate of stomatal development

The Somatic Embryogenesis Receptor Kinase (SERK) family of receptor-like kinases (RLKs), which comprises five members (SERK1-SERK5), also plays essential roles in stomatal development. SERK5 is likely a nonfunctional kinase, and knockout of the remaining four members (*serk1/serk2/serk3/serk4*) results in embryonic lethality. Analysis of the stomatal phenotypes of *serk* mutants revealed that stomatal developmental defects were exclusively observed in the *serk1*/*serk2*/*serk3* (*bak1*) triple null mutants, but not in single, double, or other triple null mutants (Meng et al., 2015). Notably, the *serk1*/*serk2*/*serk3* triple mutants exhibited not only stomatal clusters but also growth morphology similar to that of the *er erl1 erl2* triple mutants (**Table 1**) (Meng et al., 2015). As expected, SERKs physically interact with both ERf receptors and TMM. Furthermore, transphosphorylation of the ER-SERK3 receptor complex was detected, suggesting that SERK-mediated stomatal development signals may be transduced through transphosphorylation with ERf receptors (Meng et al., 2015). These results indicate that SERKs form a multiprotein receptorsome with ERf receptors and TMM to regulate stomatal development (Giannoutsou et al., 2025; Ding et al., 2025; Chen and Torii, 2023). In addition, SERKs can form constitutive, ligand-independent complexes with BIR3 (BAK1-INTERACTING RECEPTOR-LIKE KINASE3) LRR ectodomains, which regulate stomatal development, while BIR3 negatively modulates LRR-RK signaling (**Figure 1**) (Hohmann et al., 2020).

## 5 The TMM-ERf-SERKs receptorsome recognizes EPF/EPFLf ligands and activates downstream YDA-MAPK cascade

Several EPF/EPFL peptides have been shown to be recognized by the TMM-ERf-SERKs receptorsome (**Table 1**). EPF1, the first identified ligand for these receptors in stomatal development, is specifically expressed in late Ms, GMCs, and GCs (Hara et al., 2007). Its overexpression causes numerous Ms to fail to differentiate into GMCs, thereby significantly repressing stomatal production. In contrast, loss-of-function *epf1* mutants exhibit clustered stomata, indicating that EPF1 is essential for maintaining the one-cell-spacing rule of stomatal patterning (Hara et al., 2007). EPF2 is specifically expressed in early stomatal lineage cell including MMCs and Ms (Hara et al., 2009; Hunt and Gray, 2009). Overexpression of *EPF2* inhibits entry into the stomatal lineage, leading to a drastic reduction in stomatal formation. Conversely, knockout of *EPF2* increases the number of cells undergoing asymmetric division, producing numerous small epidermal cells that express stomatal lineage markers (Hara et al., 2009; Hunt and Gray, 2009). Detailed crystal structure analyses of ligand-receptor complexes and ligand-receptor binding experiments have confirmed that both EPF1 and EPF2 are recognized by constitutive complexes of TMM with ER or ERL1 (Lin et al., 2017; Kim and Torii, 2024; Chen et al., 2025b). Additionally, EPF2 and EPF1 induce the association of ER with SERKs and ERL1 with SERKs, respectively (**Figure 1**) (Meng et al., 2015; Herrmann and Torii, 2021; Giannoutsou et al., 2025).

EPFL6/CHALLAH is expressed in the endodermis of hypocotyls, inflorescence stems, and pedicels, and is secreted to the epidermis to modulate stomatal development (Abrash and Bergmann, 2010). Loss-of-function mutations in *EPFL6* can rescue the phenotype of absent stomata on the hypocotyls of *tmm* mutants. Furthermore, overexpression of *EPFL6* completely inhibits stomatal formation in *tmm* mutants but only partially suppresses it in wild-type plants. These observations suggest that TMM counteracts the inhibitory effects of EPFL6 (Abrash and Bergmann, 2010). Moreover, loss of ERf receptors alleviates the overexpression phenotypes of *EPFL6* in the *tmm* background, indicating that EPFL6 suppresses stomatal development primarily in an ERf receptor-dependent manner (Abrash and Bergmann, 2010). EPFL6 has two paralogs, EPFL4 and EPFL5, which function redundantly with EPFL6 (Abrash et al., 2011). In contrast to EPF1/2, detailed crystal structure analyses and ligand-receptor binding experiments have demonstrated that EPFL4/6 can be recognized by ERf receptors alone, and their interaction with ERf is significantly reduced in the presence of TMM (**Figure 1**) (Lin et al., 2017; Giannoutsou et al., 2025).

EPFL9/STOMAGEN is expressed in mesophyll cells and secreted to the epidermis to regulate stomatal development (Hunt et al., 2010; Kondo et al., 2010; Sugano et al., 2010). In contrast to EPF1/2 and EPFL6, overexpression of *EPFL9* promotes stomatal formation, while knockout of *EPFL9* inhibits it, indicating that EPFL9 acts as a positive regulator of stomatal development. EPFL9 has been shown to compete with EPF1/2 for binding to the ERf-TMM complex (Lee et al., 2015).

Downstream of the ligand-receptor complex lies a mitogen-activated protein kinase (MAPK) cascade composed of YODA (YDA)/MAPKKK, four MAPKKs (MKK4/5/7/9), and two MAPKs (MPK3/6) (**Table 1**) (Bergmann et al., 2004; Wang et al., 2007; Lampard et al., 2009; Ding et al., 2025). Perception of EPF1/2 by the TMM-ERf-SERK receptor complex triggers the phosphorylation of MPK3/6, which in turn phosphorylates SPCH-SCRM (Lampard et al., 2008; Putarjunan et al., 2019). This phosphorylation event leads to the degradation of SPCH-SCRM (Putarjunan et al., 2019), thereby repressing stomatal formation (**Figure 1**). However, binding of EPFL9 to the receptor complex does not activate the YDA-MAPK cascade, which relieves the inhibitory effects of EPF1/2 on stomatal production and thus promotes stomatal development (Lee et al., 2015). Nevertheless, how signals mediated by this ligand-receptor complex are transmitted to the YDA-MAPK cascade remains largely unclear.

## 6 Regulation of stomatal development by receptor-like kinase HAESA-like 1, CLAVATA1 and MUSTACHES

The LRR-RLKs *HSL1*, *CLV1*, and *MUS* also play crucial roles in stomatal development (**Table 1**) (Keerthisinghe et al., 2015; Qian et al., 2018; Blümke et al., 2021; Chen et al., 2023). *HSL1* is widely expressed in the leaf epidermis, and its loss-of-function mutants exhibit enhanced division activity of leaf epidermal cells, resulting in increased numbers of both guard cells (GCs) and non-GC cells (Qian et al., 2018). HSL1 can recognize the ligands CLAVATA3/ESR-RELATED 9/10 (CLE9/10), which are preferentially expressed in stomatal lineage cells and act to inhibit the proliferation of these cells. Additionally, HSL1 is capable of recognizing ligands CLE8, CLE13, and CLE14, and recruits SERKs as co-receptors (**Figure 1**) (Qian et al., 2018). *CLV1* is well-known for its key role in regulating stem cells in both root and shoot meristems. It is also highly expressed in stomatal lineage cells, and mutation of *CLV1* leads to the formation of clustered stomata (Blümke et al., 2021). Furthermore, CLV1 has been shown to interact with the membrane-associated receptor-like cytoplasmic kinase MAZZA (MAZ). Compared with *clv1* single mutants, the additional mutation of *MAZ* in the *clv1* background further increases the number of clustered stomata, indicating that the CLV1-MAZ signaling module is involved in the modulation of stomatal patterning (**Figure 1**) (Blümke et al., 2021). Loss of *MUS* function causes the formation of GCs with disrupted bilateral symmetry and skewed stomatal pores. This phenotype may be attributed to the impairment of the bilateral symmetry of radial microtubule arrays, as well as the disruption of microtubule growth and polarity in guard cells (Keerthisinghe et al., 2015). MUS-GFP is localized at both division sites and the cell periphery throughout seedling development. However, in the leaf epidermis, strong peripheral MUS-GFP expression is exclusively observed in guard mother cells (GMCs) of the stomatal lineage. Notably, during the symmetric division of GMCs, MUS-GFP is absent from the cell plate and phragmoplast (Keerthisinghe et al., 2015). Collectively, these results suggest that MUS contributes to stomatal formation by regulating cell wall biosynthesis and maintaining the cytoskeletal polarity of GMCs and GCs.

## 7 The receptor-like kinases involved in stomatal development in grasses

The EPF-TMM-ERECTA module has been proposed to represent a conserved, universal patterning system across the plant kingdom (Caine et al., 2016). However, the roles of homologs of the aforementioned RLKs and RLPs in grass stomatal development remain largely elusive to date. During SC formation in maize, two additional RLKs, designated PAN-GLOSS1 (PAN1) and PAN2, are localized polarly on the SMC membrane at the contact interface between SMCs and GMCs; notably, the polar localization of PAN1 is dependent on PAN2 (Cartwright et al., 2009; Zhang et al., 2012; Cheng and Raissig, 2023). These two RLKs function redundantly to regulate the asymmetric division of SMCs: loss-of-function mutations in either result in abnormal polar division of SMCs, ultimately producing aberrant SCs (**Figure 2**) (Liu et al., 2024). Mechanistically, PAN1 interacts with ROP2 (Rho-related GTPase from Plants 2) and ROP9-proteins that also exhibit polar localization on the SMC membrane at the SMC-GMC contact surface-to regulate SMC asymmetric division (Humphries et al., 2011). Additionally, the SCAR/WAVE (Suppressor of cAMP Receptor defect/WASP-family Verprolin-homologous protein) complex is essential for the polar localization of PAN2 (Facette et al., 2015; Berg and Raissig, 2025).

## 8 Conclusion and unresolved questions

Several LRR-RLKs and LRR-RLPs, including TMM, ERf, SERKs, HSL1, CLV1, MUS, and PAN1/2, have been identified as stomatal receptors that are responsible for perceiving both developmental and environmental cues during stomatal development. However, their specific roles in this process remain to be investigated in more depth, and numerous unresolved questions persist in this field. Generally, upon perceiving extracellular ligand signals, RLK receptors form heterodimers with co-receptors in the plasma membrane, which triggers autophosphorylation and activation of the receptor’s intracellular kinase domain. Subsequently, signals are transduced intracellularly through a cascade of phosphorylation events, enabling plant cells to respond to extracellular stimuli. Nevertheless, these detailed mechanisms underlying stomatal receptors remain largely unclear.

Most stomatal receptors exhibit specific expression patterns in stomatal lineage cells relative to their promoter activity, implying that they are subject to post-translational regulation. Despite reports that two closely related plant U-box ubiquitin E3 ligases, PUB30 and PUB31, are involved in regulating ER ubiquitination and degradation (Chen et al., 2023), little is known about the overall post-translational regulatory mechanisms of these receptors. At the transcriptional level, DNA hypermethylation mediated by the histone demethylase IBM1 has been shown to regulate the expression of *ERf* genes (Wang et al., 2016), and *TMM* and *ERf* kinases are direct targets of *SPCH*. Clarifying the regulatory mechanisms governing the expression of these receptors is therefore indispensable for elucidating their critical roles in stomatal development.

Furthermore, the ligands of CLV1, MUS, and PAN1/2 in stomatal development have not yet been identified. This is particularly true for PAN1/2, as it has long been hypothesized that these receptors recognize specific ligands derived from GMCs to induce SC formation. Finally, research on genes involved in stomatal development in *Arabidopsis* provides a valuable foundation for investigating whether their orthologs play conserved roles in stomatal development in grasses. Notable examples include key transcription factors governing stomatal cell fate specification and the EPFL family of small secreted peptides, which modulate stomatal density. However, the functional roles of receptors such as TMM and ERf homologs in grass stomatal development remain to be elucidated. Importantly, stomata in *Arabidopsis* are distributed irregularly across the leaf epidermis, whereas in grasses, they are arranged in parallel files flanking leaf veins on both epidermal surfaces. In addition, the processes of stomatal development in grasses differ considerably from those in *Arabidopsis*. This suggests that homologs of *Arabidopsis*-identified stomatal regulators may exert distinct functions in grasses. For example, the key transcription factor MUTE is predominantly expressed in GMCs in both *Arabidopsis* and grasses; however, only grass MUTE exhibits intercellular mobility and can move to neighboring cells to induce SC formation. Therefore, grass systems may reveal receptor functions that are less evident in *Arabidopsis*, a finding that is crucial for understanding the evolutionary trajectory of stomata across plants.
